# Motor Unit Behaviour in the Biceps Brachii During Asymmetric Bilateral Versus Unilateral Dynamic Force Tracking Across Force Levels and Rates of Force Development: A Preliminary Study

**DOI:** 10.3390/life16091404

**Published:** 2026-08-25

**Authors:** Jianchang Ren, Fang Qiu, Liyun Ma, Haili Xiao, Zhangzhi Jia

**Affiliations:** 1 Institute of Sport and Health, Lingnan Normal University, Zhanjiang 524048, China; renjc@lingnan.edu.cn; 2 Guangdong Provincial Kay Laboratory of Development and Education for Special Needs Child, Lingnan Normal University, Zhanjiang 524037, China; 3 School of Physical Education & Health, Shanghai University of International Business and Economics, Shanghai 201620, China; 4 Guangdong Huangcun Sports Training Center, Guangzhou 510663, China

**Keywords:** electromyography, motor unit, common synaptic input, recruitment threshold

## Abstract

Most bilateral actions in daily life are asymmetric: one limb sustains a steady postural load while the other performs a continuously adjusted force task. Whether the influence of such a sustained contraction on the contralateral limb is a fixed property of bilateral coordination or a strategy that higher centres rescale with task demand is unknown. In this exploratory study, we examined motor unit recruitment and common neural drive in the biceps brachii during asymmetric bilateral versus unilateral isometric force tracking, tested whether the interlimb effect depends on force level and rate of force development. Fifteen healthy adults tracked trapezoidal force profiles with the right biceps brachii at two force levels (10% and 30% of maximal voluntary contraction) and three rates of force development (5%, 10%, and 20% per second), performed unilaterally and bilaterally while the left biceps brachii held a constant 20% contraction. High-density surface electromyography was decomposed into individual motor units. Relative to unilateral tracking, the bilateral condition tended to raise recruitment threshold, lowered steady-state discharge rate, increased common-input variability, and reduced force stability, whereas action-potential amplitude and the variance explained by the common input (41–45%) were unchanged. No force- or rate-dependent interactions emerged. Although consistent with a spinal constraint, these findings cannot exclude cortical strategies and should be interpreted cautiously given the small sample.

## 1. Introduction

Bilateral upper limb actions in daily life rarely involve symmetric, constant, or uniformly paced effort from both arms. Common daily tasks include opening a jar, carrying a bag while manipulating an object, or stabilizing one limb as the other performs a precise movement. These tasks share a defining feature: the two limbs simultaneously sustain qualitatively different force demands. Typically, one limb maintains a relatively stable postural load, while the other executes dynamically modulated contractions. Understanding the neural control of bilateral coordination therefore requires moving beyond the question of what happens when both hands exert force simultaneously under matched conditions, and toward how sustained contraction in one limb modifies the ongoing, intensity-varying output of the other [1].

At the cortical level, the distinction between unilateral and bilateral motor representations is well established. Electrophysiological recordings in non-human primates have shown that neurons across cortical motor areas differentially modulate their discharge during unimanual versus bimanual tasks [2,3,4,5], and that excitatory transcallosal drive is suppressed when both limbs act simultaneously [6]. The behavioral consequences of disrupting this organization are telling: a unilateral lesion confined to the supplementary motor area leaves general limb movements largely intact yet produces enduring impairments selectively in bimanual coordination [7,8]. Translating these observations to humans, neurophysiological work has documented task-specific changes in both corticospinal excitability and intracortical interactions when force is generated bilaterally rather than unilaterally [9,10], while neuroimaging has consistently revealed a broader and more intense engagement of primary sensorimotor cortex and the supplementary motor area during bilateral tasks [11,12,13,14]. Asymmetric bilateral actions place a particular demand on interhemispheric inhibition to decouple the motor outputs of the two hemispheres [15]. Recent transcranial stimulation work indicates that the magnitude of this inhibition predicts bimanual task performance and is amenable to non-invasive modulation [16,17]. Collectively, these lines of evidence converge on the conclusion that supraspinal circuits encode bilateral coordination through mechanisms qualitatively different from those engaged during unilateral movement. Yet the descending commands these circuits generate must ultimately be transformed into muscle force through spinal motoneuron pools [18], and how that transformation is shaped by bilateral constraint, at the resolution of individual motor units, has received comparatively little attention.

Decomposition of high-density surface electromyographic signals now permits non-invasive tracking of large numbers of individual motor units within a single muscle, offering a direct window onto spinal neural strategies [19,20]. Bao et al. [21] applied this approach to the first dorsal interosseous muscle. They found that bilateral isometric contractions were accompanied by reduced motor unit discharge rates, elevated recruitment thresholds, and increased variability of both force output and the first common component of neural drive. However, action potential morphology remained unchanged. That study provided the first motor unit–level evidence that bilateral constraint alters spinal neural drive and linked this alteration to changes in low-frequency common synaptic input. Comparable reductions in bilateral neural drive at maximal contraction intensities, the so-called bilateral deficit, have been documented repeatedly in lower-limb muscles as well [22,23].

The existing motor unit evidence has, however, been built almost exclusively on symmetric, Isometric, single-force-level paradigms. This leaves a mechanistically consequential question unanswered: is the bilateral constraint a fixed neural property that is independent of task intensity, or an adaptable strategy that supraspinal centers recalibrate as task demands change? We frame this question around the two most plausible, but not mutually exclusive, contributing levels of the neuraxis. On the one hand, supraspinal circuits could regulate the constraint in a task-adaptive manner, such that its magnitude scales as force demand or the rate of force development increases and coordination complexity rises; this would be expected to produce a force × side or rate × side interaction in motor unit behaviour. On the other hand, the constraint could instead reflect an integration process (plausibly, though not necessarily, spinal in origin) whose gain is comparatively insensitive to these task parameters, in which case the interlimb difference should remain approximately stable across the force–rate space.

We emphasise, however, that these two accounts are not cleanly separable by interaction testing alone. A stable interlimb effect need not be spinal: a descending command that is itself invariant across task levels would produce the same pattern [24,25]. Conversely, a scaling interlimb effect need not be cortical: spinal integration (through mechanisms such as persistent inward currents or reciprocal and recurrent inhibition) may itself vary with force or task demand. We therefore treat the presence or absence of an interaction not as a decisive test that assigns the mechanism to a single level, but as constraining evidence about whether the interlimb effect scales with task parameters—a functional property that any candidate mechanism, cortical or spinal, must ultimately accommodate. These two possibilities cannot be distinguished by any single-force, single-rate design; resolving them requires a systematic parametric examination in which bilateral–unilateral differences are tested across a force–rate space.

Force level and contraction rate are the two fundamental task dimensions that regulate motor unit behavior. Discharge rates increase with force and exhibit rate-dependent modulation during isometric force-tracking task, as recently characterized in detail for the biceps brachii and other forearm flexors [26,27,28]. The low-frequency common synaptic input to motor neurons, the principal neural determinant of force steadiness, likewise varies with task conditions in its amplitude and temporal variability [29,30,31]. Given that both force and rate independently shape motor unit recruitment and common drive, whether they also modulate the magnitude of the bilateral constraint is a question that can in principle be answered experimentally, yet has not previously been tested.

The present study addressed this question using an asymmetric bilateral isometric trapezoidal contraction paradigm designed to capture the structure of naturalistic bilateral actions. Participants maintained a constant 20% MVC isometric contraction with the left biceps brachii, simulating a sustained postural load, while the right biceps brachii tracked a trapezoidal force profile across two force levels (10% and 30% MVC) and three rates of force development (5%, 10%, and 20% MVC/s). High-density surface electromyography decomposition was used to quantify recruitment thresholds, steady-state discharge rates, and action potential peak-to-peak amplitude in the right biceps brachii, alongside principal component analysis of motor unit firing sequences to extract common synaptic input variability (PC1-CV) and concurrent force output variability (Force-CV). The primary hypothesis was that bilateral constraint would elevate recruitment thresholds, reduce discharge rates, and increase variability of common drive and force output relative to the matched unilateral condition. Critically, we tested whether the magnitude of this interlimb effect varied with force level or rate of force development, that is, whether significant force × side or rate × side interactions would emerge. Should the interlimb effect prove approximately invariant across the force–rate space examined, this would be consistent with a constraint whose gain is not readily recalibrated by the tested task parameters. We interpret such a pattern as one plausible signature of a predominantly spinal integration process. However, because the present design provides no direct neurophysiological measure of cortical, corticospinal, or spinal activity, this interpretation is offered as a hypothesis to be tested by future work. Such future work could employ techniques such as transcranial magnetic stimulation or reflex conditioning, rather than treating this as an established localisation. This distinction nonetheless carries practical relevance for how training intensity and movement speed might be considered when designing bilateral motor rehabilitation programmes [32,33].

## 2. Materials and Methods

### 2.1. Subjects

Fifteen participants (13 males and 2 females), aged between 18 and 22 years, were recruited for this study. All participants reported no history of neuromuscular disorders and provided written informed consent prior to participation. The study protocol, including the informed consent procedure, was conducted in strict accordance with the Declaration of Helsinki and approved by the Ethics Committee of Lingnan Normal University (approval number: 2023126).

Participants were recruited from the university student population via campus notices and word of mouth. All were healthy young adults (18–22 years; [13 men and 2 women; final analysed sample *n* = 14 after exclusion of one participant]) with no self-reported history of neurological, musculoskeletal, or cardiovascular disorder and no upper-limb injury in the preceding six months. All were recreationally active but not engaged in systematic resistance training of the upper limbs, and none were competitive athletes. Handedness was assessed with the Edinburgh Handedness Inventory, and all participants were confirmed right-hand dominant; the right (dominant) arm served as the tracking limb.

Participants were instructed to refrain from strenuous or unaccustomed exercise for 48 h and from caffeine, tea, and other stimulant beverages for 24 h before testing, to obtain a normal night’s sleep (≥^7^ h), and to arrive neither fasted nor immediately post-prandial (having eaten a light meal ≥^2^ h beforehand) and adequately hydrated. No participant reported use of medication, ergogenic aids, or nutritional supplements known to affect neuromuscular function. All sessions were conducted in a temperature-controlled laboratory ([22 ± 1 °C], relative humidity [~45–55%]) at a consistent time of day ([e.g., 09:00–12:00]) to limit diurnal and environmental variability.

### 2.2. Experimental Procedure

**Figure 1:**
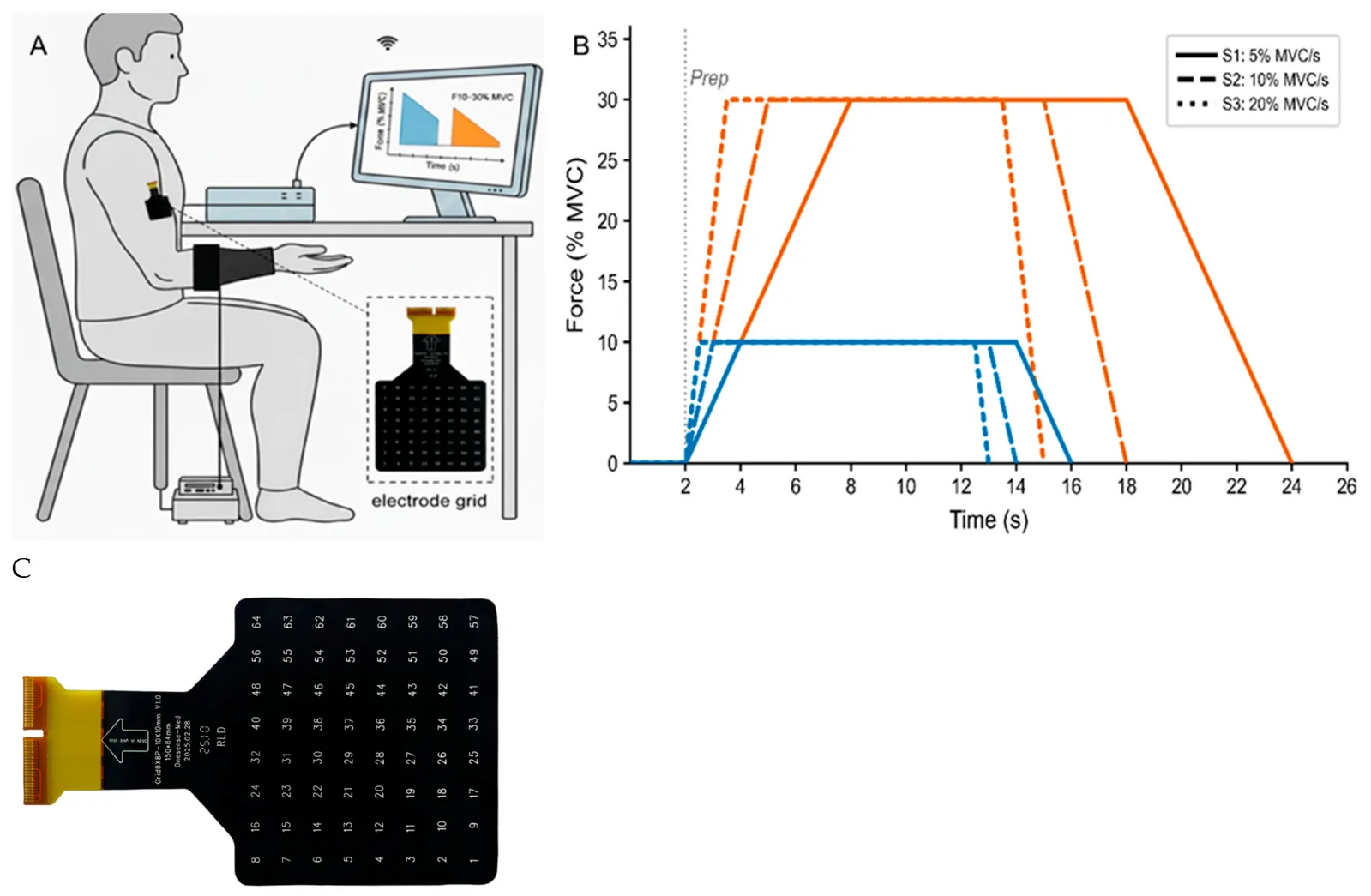
Experimental setup. (**A**) illustrates a schematic diagram of the experimental apparatus. (**B**) illustrates the 6 target force trajectories that participants were instructed to track. (**C**) electrode grid.

Participants were seated in a chair with both arms naturally at rest, elbows flexed at 90°, and palms facing upward. The midpoint of the forearm was connected to a force sensor via a load cell attachment strap to measure the force exerted by participants. Maximal voluntary contraction (MVC) was assessed separately for the right and left arms. Each participant completed two experimental conditions: unilateral contraction (right biceps tracking trapezoidal force trajectories at 10% and 30% MVC with the left arm relaxed) and bilateral contraction (right biceps tracking trapezoidal force trajectories at 10% and 30% MVC while the left biceps maintained a constant 20% left-arm-MVC contraction imposed by a fixed external load rather than by visual force feedback; because this load was mechanically constant, the contralateral contraction level was held invariant across all trials by design). The two conditions were performed within a single session on the same day, in randomised order, separated by a minimum interval of 30 min. At the beginning of the experiment, participants performed 2–3 MVC trials, each lasting 3–5 s with an inter-trial interval of 40–60 s, with verbal encouragement and visual feedback. The average of the 2–3 MVC trials was used as the MVC value. Participants then tracked trapezoidal force trajectories by controlling elbow-flexion force. Each trajectory comprised a rising phase (force increasing linearly from rest to the target), a plateau phase (target force held steady), and a falling phase (force decreasing linearly to rest). Trajectories were defined by 2 force levels (10% and 30% MVC) and 3 rates of force development (S1: 5% MVC/s; S2: 10% MVC/s; S3: 20% MVC/s), yielding 6 distinct trajectories (Figure 1B). The order of the six trajectories was randomised for each participant within each condition.

For each condition, participants performed 2 repeated trials of each of the 6 trajectories. For each trajectory, only the higher-quality repetition (based on decomposition quality and force-tracking accuracy) was retained for analysis, so that each trajectory contributed a single analysed trial per participant. Each trial lasted 13–24 s, with a 30–45 s rest between consecutive trials to minimise fatigue. Throughout, both the target trajectory and the participant’s generated force were displayed in real time as visual feedback (for the right, tracking arm).

### 2.3. Force Signal Acquisition

During the experiment, participants were comfortably seated facing the apparatus. Participants extended their dominant arm forward, maintaining the elbow at approximately 90°, with the midpoint of the forearm consistently aligned with the force sensor (Figure 1A). To minimize unwanted movement, the elbow was stabilized with a strap, and participants were instructed to primarily activate the upper arm musculature during force generation. A graphical user interface developed in MATLAB R2022a enabled real-time data acquisition from the force sensor via a serial connection and provided continuous visual feedback to participants. Force signals were recorded using a DYLY-103 S-type tension-and-compression load cell (capacity: 1000 N; sensitivity: 2.0 ± 10% mV/V; zero output: ±1% F.S.; nonlinearity: 0.03% F.S.; Bengbu Dayang Sensing System Engineering Co., Ltd., Bengbu, China) operating at a sampling rate of 2000 Hz.

### 2.4. High-Density Surface Electromyography Recording

A pair of 64-channel electrode arrays (8 × 8 configuration; Grid8X8P-10 mm, Onesense, Shanghai, China) was employed to capture high-density sEMG signals. Each electrode had a diameter of 3 mm with an inter-electrode spacing of 10 mm along both axes (Figure 1A). The electrode arrays were affixed over the muscle belly of the right biceps brachii. Data from each array were fed into a multi-channel signal acquisition interface, which relayed the information wirelessly via Wi-Fi to a receiver station interfaced with a computer (Oct-HD, Onesense, China).

Surface EMG was recorded from the right biceps brachii using a 64-channel (8 × 8) high-density electrode grid with a 10 mm inter-electrode distance. The grid was positioned over the muscle belly with the electrode columns aligned to the muscle-fibre direction, between the innervation zone and the distal tendon. The skin was shaved, lightly abraded and cleaned with alcohol, and conductive paste was applied to each electrode cavity to reduce contact impedance. A reference electrode was placed over the ulnar styloid process. Channels showing artefact, saturation or poor skin contact were rejected by visual inspection before decomposition.

The sEMG acquisition was conducted in monopolar mode with a gain setting of 1000 and a sampling rate of 2000 Hz. Raw signals were processed through hardware-level bandpass filtering (3–900 Hz) and converted to digital form using a 24-bit analog-digital converter. Real-time monitoring and archival of signals were managed through the Oct-HD PC Client software 0.13 suite, with all recordings retained for subsequent offline analysis. A custom synchronization protocol based on an Arduino microcontroller platform (Arduino AG, Monza, Italy) was constructed to align the force and sEMG data streams. Synchronization trigger signals were issued by the microcontroller at the beginning and end of each experimental trial and transmitted to the sEMG receiver to maintain temporal alignment precision.

### 2.5. sEMG Decomposition

**Figure 2:**
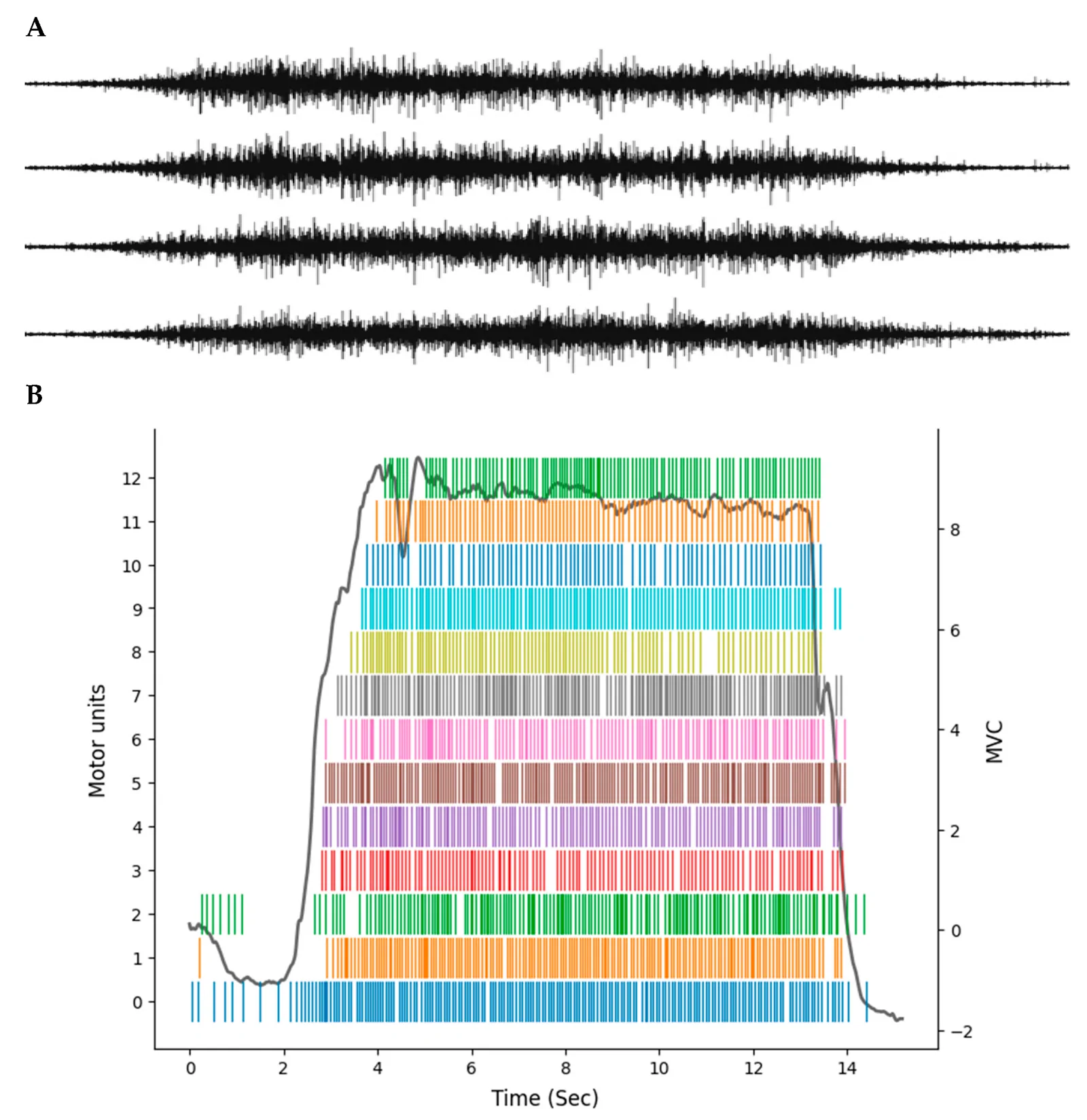

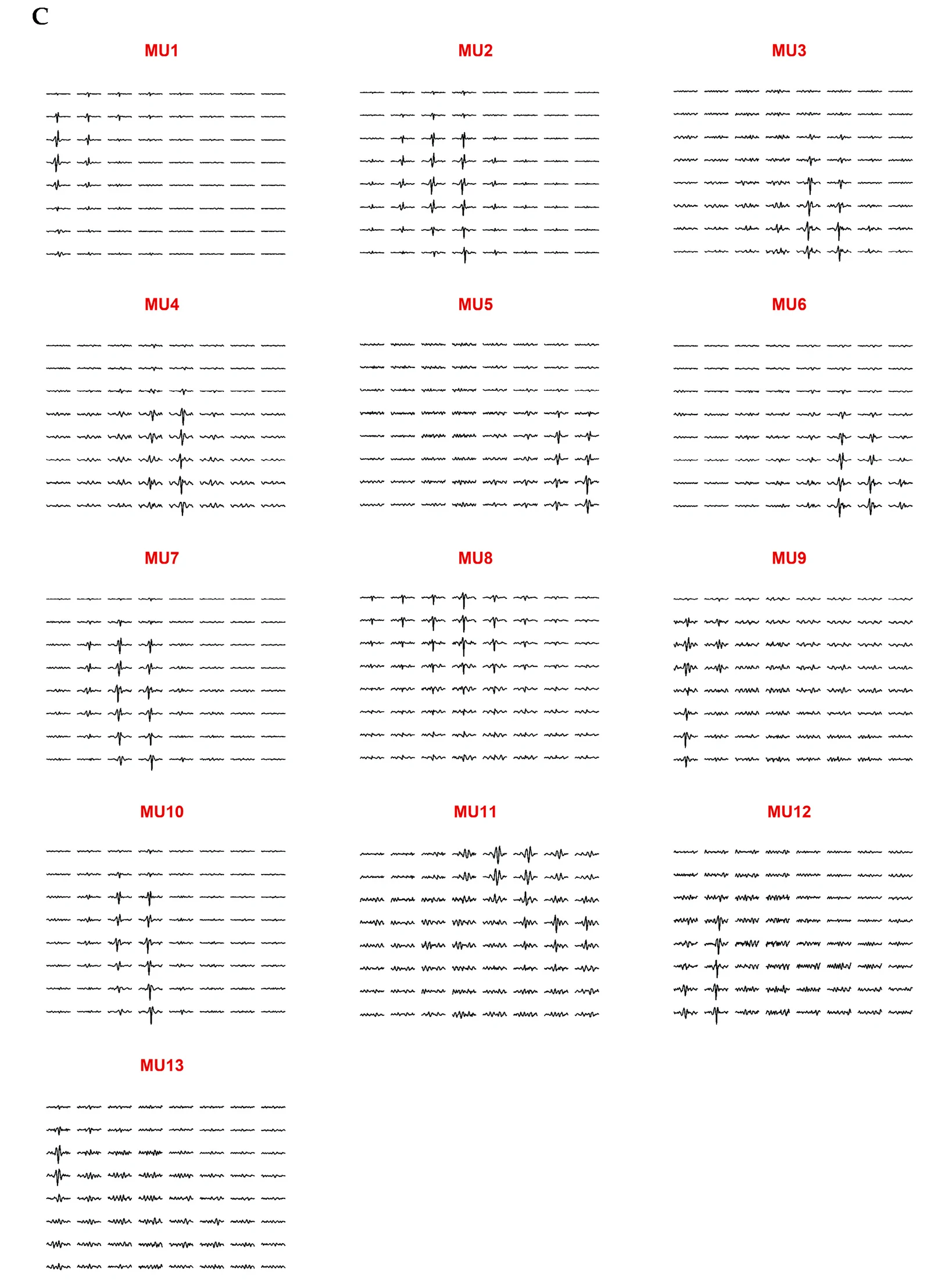
sEMG signal decomposition and motor unit identification. (**A**) Raw multi-channel sEMG signals recorded during a voluntary contraction. (**B**) Motor unit discharge raster plot of the identified units following decomposition and multi-stage quality filtering. Each row of vertical ticks represents the firing events of a single motor unit across the contraction period. (**C**) Motor unit action potential (MUAP) waveforms extracted for each identified unit (MU1–MU13). Each panel shows the superimposed action potentials of a single motor unit across multiple firings, illustrating the consistency of waveform morphology.

Multi-channel surface electromyography (sEMG) signals (Figure 2A) were decomposed into motor unit discharge sequences through a combined approach of time-delay embedding and blind source separation. The method treats multi-channel sEMG as a convolutional mixture of motor unit discharge patterns. First, time-delay embedding was applied to expand the spatial dimensionality of the original signal using a delay parameter r (for example, r = 3 for 64-channel recordings expands the observation matrix to 256 dimensions), enhancing the separability of the convolutional mixture. Subsequently, each expanded channel was mean-centered, and principal component analysis (PCA) was employed to whiten the signals, reducing inter-channel correlations and facilitating independent component analysis.

The decomposition algorithm iteratively applied FastICA to the whitened expanded sEMG signals to extract candidate independent components. Separation vectors were randomly initialized and iteratively updated via the FastICA fixed-point algorithm (convergence threshold 10^−4^, maximum iteration limit 500) until convergence, with this procedure repeated num_iter times to generate multiple candidate components. For each candidate source sequence, an energy-based peak detection method was used to identify discharge times: the source sequence was squared to enhance peak visibility, local maxima were identified, and clustering analysis partitioned peak amplitudes into high and low categories, with positions of high-amplitude peaks designated as discharge times. Candidate components were subsequently subjected to multi-stage quality screening to retain reliable motor units. First, physiological discharge rate screening ensured average discharge rates within the 4–35 pulses/second range. Second, artifact and redundancy detection merged pulses with inter-spike intervals less than 20 ms, and identified and removed temporally delayed duplicate motor units (maximum delay window: 10 ms; synchronization threshold: 0.5). Third, silhouette coefficient (SIL) evaluation was performed via low-pass filtering (cutoff frequency: 500 Hz), peak clustering, and average silhouette value calculation. Components with SIL coefficients greater than 0.9 were retained, while those with insufficient peak separability were excluded. Finally, morphological screening excluded components exhibiting abnormal pulse half-width, prolonged high-percentile peak width, excessive energy concentration ratio, or discharge patterns deviating from typical narrow motor unit action potentials. Decomposition was performed with a fully automated, fixed-threshold pipeline, and discharge instants were not manually edited; the only operator-dependent step was the pre-decomposition visual rejection of unusable channels. Decomposition yielded 1111 motor units across the 14 participants; per-condition yields and their statistical comparison are reported in Supplementary Tables S1–S4, and mean decomposition quality (SIL = 0.93; steady-state COVisi ≈ 24.5%) is reported by side in Table S5. Motor-unit yield did not differ significantly across side, force or rate (all *p* > 0.05). The retained motor units were presented as a discharge raster plot (Figure 2B), with waveform templates for individual motor units displayed in Figure 2C, demonstrating successful isolation of motor unit action potential morphologies from the original multi-channel sEMG signals. The final dataset contained the discharge times, average discharge rates, recruitment and de-recruitment period discharge rates, and waveform morphological characteristics for each motor unit, which were subsequently used for analysis of motor control characteristics at different force levels and rates of force development.

### 2.6. Recruitment Threshold

The recruitment threshold (RT) was defined as the mean force level (%MVC) corresponding to the first four motor unit discharge events during the ascending phase. Specifically, within the time window from the onset of force rise to the initiation of the plateau phase, the discharge pulse sequence of each motor unit was identified. The force levels (%MVC) corresponding to the first four discharge pulses were averaged to obtain the recruitment threshold for that motor unit.

### 2.7. Plateau Phase Discharge Rate

The plateau phase discharge rate was calculated based on the time window of the plateau phase (from plateau onset to the initiation of force decline). All discharge pulses of the motor unit occurring within the plateau phase were identified. The time intervals between consecutive discharges were computed, referred to as interspike intervals (ISI) in seconds. The instantaneous discharge rate sequence was obtained by taking the reciprocal of each ISI (IDR = 1/ISI). To exclude physiologically implausible values, instantaneous discharge rates outside the range of 3–100 pulses per second were removed from the analysis. On average, 1% of all instantaneous discharge rates fell outside this range and were excluded. The arithmetic mean of the remaining valid IDR values was computed and designated as the mean discharge rate during the plateau phase for that motor unit.

### 2.8. Common Synaptic Input Analysis via Principal Component Decomposition

Muscle force output is modulated by low-frequency synaptic inputs to motor neurons. To investigate the relationship between motor unit discharge rate and force output during unilateral and bilateral contractions, we used principal component analysis (PCA) to derive a proxy for the common synaptic input to the motor-unit pool. Only trials containing at least four concurrently active motor units were entered into the PCA, because the first principal component cannot provide a stable estimate of the shared low-frequency fluctuation from fewer units.PC1 (the first principal component, taken as a proxy for the common synaptic input) was derived as follows: the instantaneous discharge rates of individual motor units were first computed from smoothed spike trains (Savitzky-Golay filter, window = 100 ms). The resulting discharge rate matrix was then linearly detrended column-wise to remove slow drift. Subsequently, each column was Z-score normalized to equalize the scale across motor units. PCA was performed on the normalized matrix, and the first principal component (PC1) was extracted as the linear combination of motor unit discharge rates that explained the maximum variance. The sign of PC1 was corrected by ensuring positive correlation with force output.

PC1-CV, the coefficient of variation of the first principal component, was computed within the plateau phase as: $CV_{PC1}=\frac{SD\left(PC1_{detrended}\right)}{mean\left(\left|PC1\right|\right)}\times \;100\%$, Because PC1 is a zero-crossing signal after Z-scoring and takes both positive and negative values, its ordinary mean approaches zero; we therefore used the standard deviation of the linearly detrended PC1 scores as the numerator, to isolate genuine plateau-phase fluctuation from slow drift, and the mean of the absolute PC1 scores as a strictly positive, robust scale factor in the denominator. The resulting ratio is a stable, dimensionless index of the relative amplitude of the common neural fluctuation.

### 2.9. Statistical Analysis

For each subject, descriptive statistics (mean and SD) of motor unit characteristics—including RT, steady-state discharge rate (DR_steady), and the coefficient of variation of the common synaptic input (CV_FCC)—were calculated separately for unilateral and bilateral contractions under all combinations of force and rate conditions.

Linear mixed-effects models (LMM) were fitted to the motor unit (MU)-level data [34]. Each model included subject as a random intercept and incorporated side (unilateral vs. bilateral; reference level: bilateral), force level (F10 vs. F30; reference level: F10), and contraction rate (V5/V10/V20; reference level: V10) as fixed-effect factors. To examine interactive effects among factors, second-order interactions (force × side, force × rate, side × rate) and a three-way interaction (force × side × rate) were included in the models. Model parameters were estimated using restricted maximum likelihood (REML) [35,36].

For each participant and condition, a single trial was retained for analysis: the trial with the higher decomposition quality, indexed by the silhouette values of the decomposed motor unit sources. Individual motor units were not tracked across the two repeated trials. Linear mixed-effects models included a by-participant random intercept; given the sample size (n = 14), the random-effects structure was kept parsimonious to avoid over-parameterisation.

To confirm that the laterality effects were not driven by non-independence of motor units within participants or by unequal numbers of units contributed across participants, we performed a participant-level sensitivity analysis. Data were aggregated to a single mean value per participant for each force × side × velocity condition, giving every participant equal weight, and the side effect within each force level was re-examined using paired *t*-tests. Statistical significance was set at α = 0.05. All analyses were performed in Python 3.11 using the MixedLM function from the statsmodels library. When interaction terms reached statistical significance, simple effects analysis was conducted to examine pairwise comparisons within specific factor levels.

## 3. Results

### 3.1. Motor Unit Decomposition Characteristics

A total of 1111 motor units were decomposed from the right biceps brachii of 14 subjects (one male subject was excluded due to excessive missing data) across all force levels and rates of force development. The average MU count per subject was 79.4 ± 28.5 (range: 37–135). Motor units were identified in unilateral contractions (41.29 ± 14.75) and bilateral contractions (38.07 ± 14.24), with no significant difference between contraction sides (*p* = 0.563). Decomposition accuracy was consistently high across contraction types (SIL; bilateral: 0.9277 ± 0.0195 vs. unilateral: 0.9294 ± 0.0200), indicating reliable motor unit identification regardless of contraction modality. Detailed statistical results for decomposition accuracy and motor unit variability indices are presented in Supplementary Tables S1–S5.

### 3.2. Recruitment Threshold and Motor Unit Action Potential Amplitude

**Figure 3:**
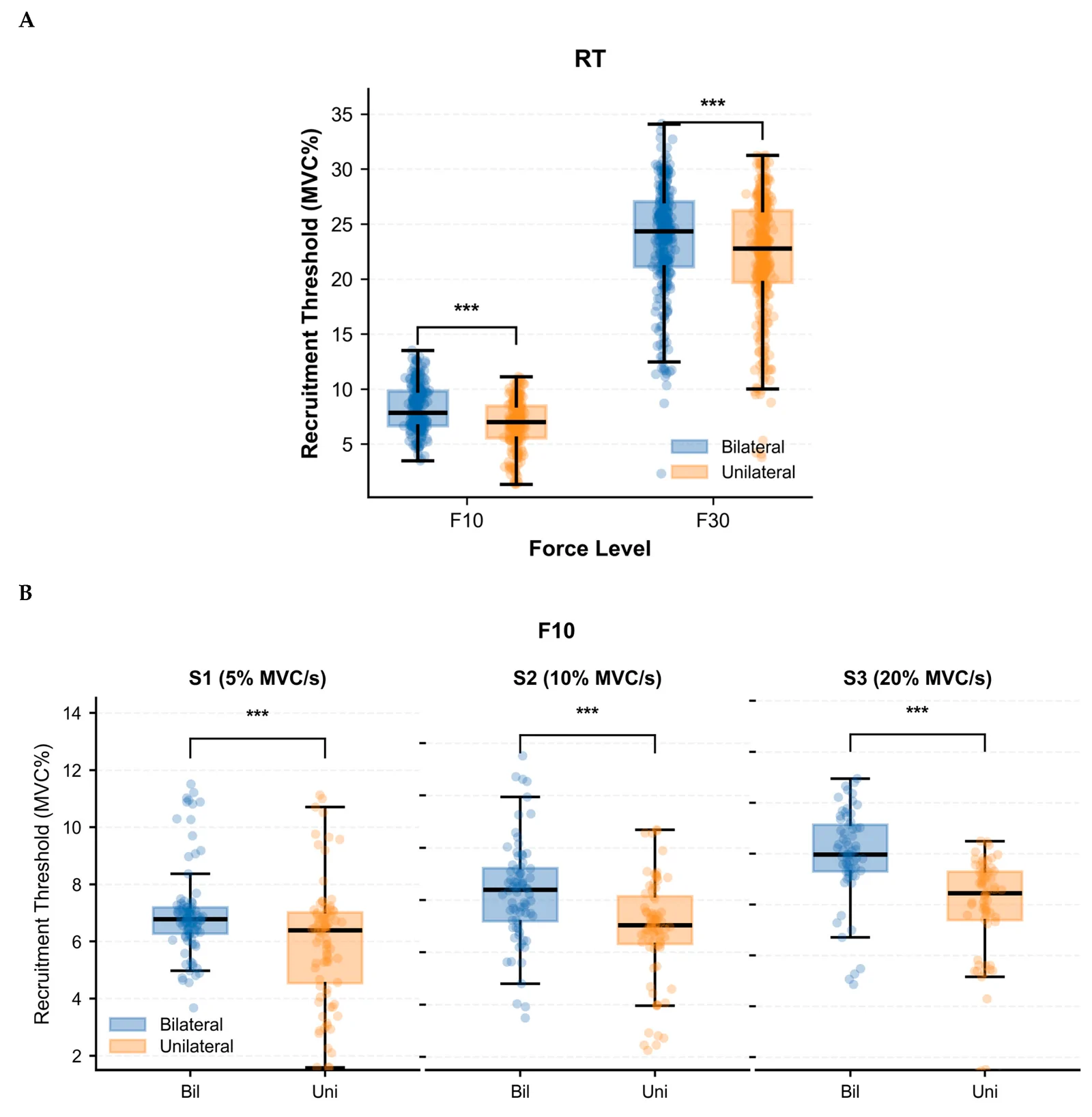

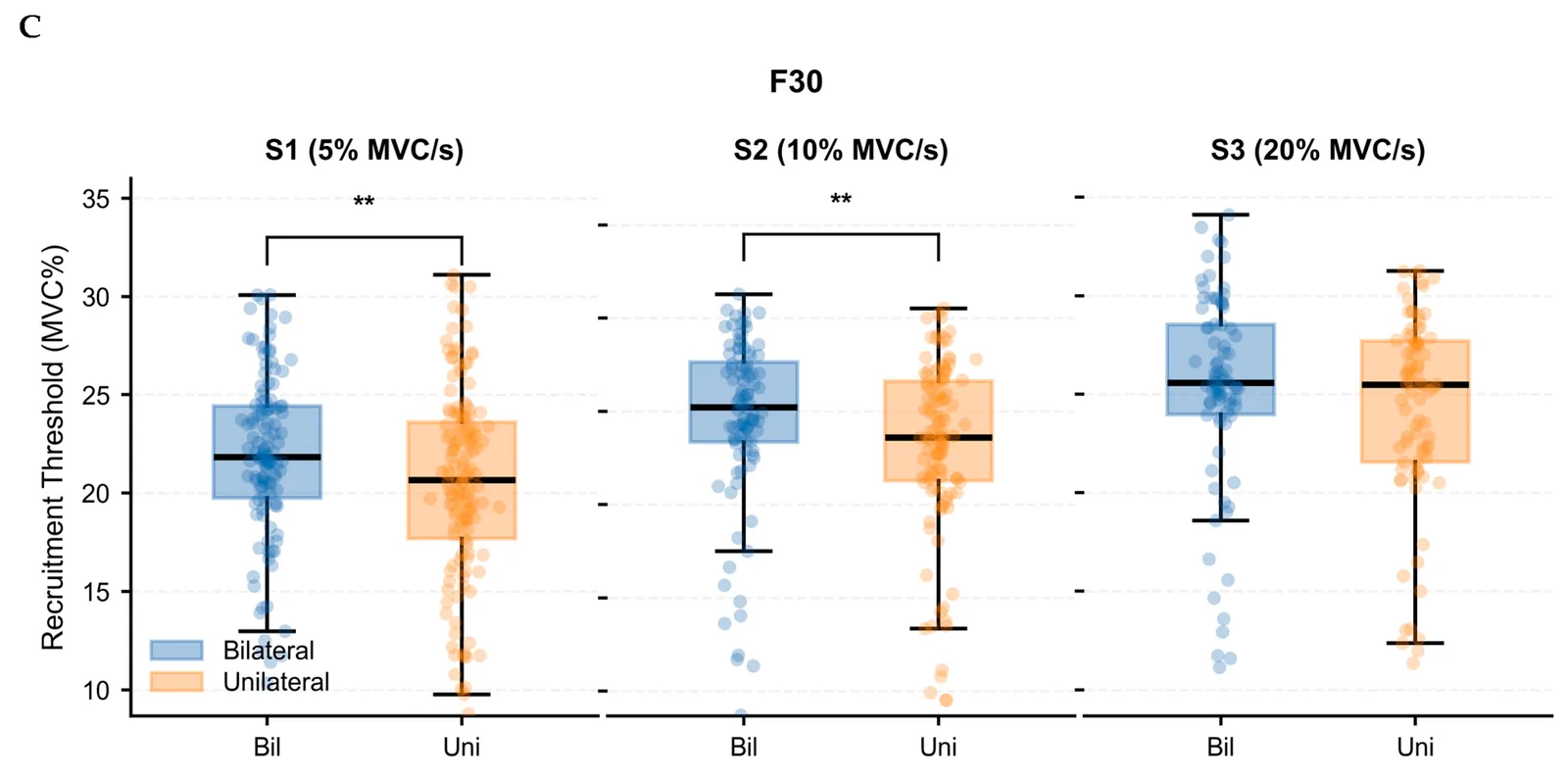
Recruitment Threshold (RT) Across Force Levels and Contraction Sides. RT expressed as percentage of maximum voluntary contraction (%MVC) across different force levels and rates of force development. (**A**) Overall comparison of RT between F10 and F30 force levels, showing bilateral (Bil, blue) and unilateral (Uni, orange) contractions. Box plots with overlaid individual data points display individual data points with median and quartiles. (**B**) RT values at F10 force level across three rates of force development (S1 [5% MVC/s], S2 [10% MVC/s], and S3 [20% MVC/s]), comparing bilateral and unilateral contractions. (**C**) RT values at F30 force level across the same three rate. Horizontal bars represent means with 95% confidence intervals. *** *p* < 0.001, ** *p* < 0.01,  for bilateral vs. unilateral contrasts within each rate level.

Linear mixed-effects modeling of recruitment threshold (RT) revealed a significant main effect of force level (F30 vs. F10: β = +14.86, SE = 0.265, z = 56.16, *p* < 0.001; Figure 3A), with RT markedly higher during F30 contractions (22.83 ± 4.99% MVC) than during F10 contractions (7.55 ± 2.46% MVC). Estimated marginal means (EMMs, marginalised with equal weights across force-ramp rate) for the four force × side conditions were: F10 bilateral 8.68% MVC (95% CI [7.82, 9.55]), F10 unilateral 7.16% MVC ([6.29, 8.03]), F30 bilateral 23.71% MVC ([22.89, 24.53]), and F30 unilateral 22.41% MVC ([21.61, 23.22]), consistent with the raw condition means shown in Figure 3B,C. Model-based contrasts confirmed that bilateral RT was significantly higher than unilateral RT at both force levels (Unilateral − Bilateral: F10, −1.52% MVC, 95% CI [−2.22, −0.83], *p* < 0.001; F30, −1.29% MVC, 95% CI [−1.89, −0.70], *p* < 0.001). The force × side interaction was not statistically significant (β = +0.23, SE = 0.47, 95% CI [−0.69, 1.15], z = 0.49, *p* = 0.623), and neither the side × force-ramp-rate nor the three-way (force × side × force-ramp-rate) interaction reached significance (all *p* > 0.05). These results indicate that the bilateral elevation of RT did not exhibit a significant interaction with force levels and force-ramp rates.

**Figure 4:**
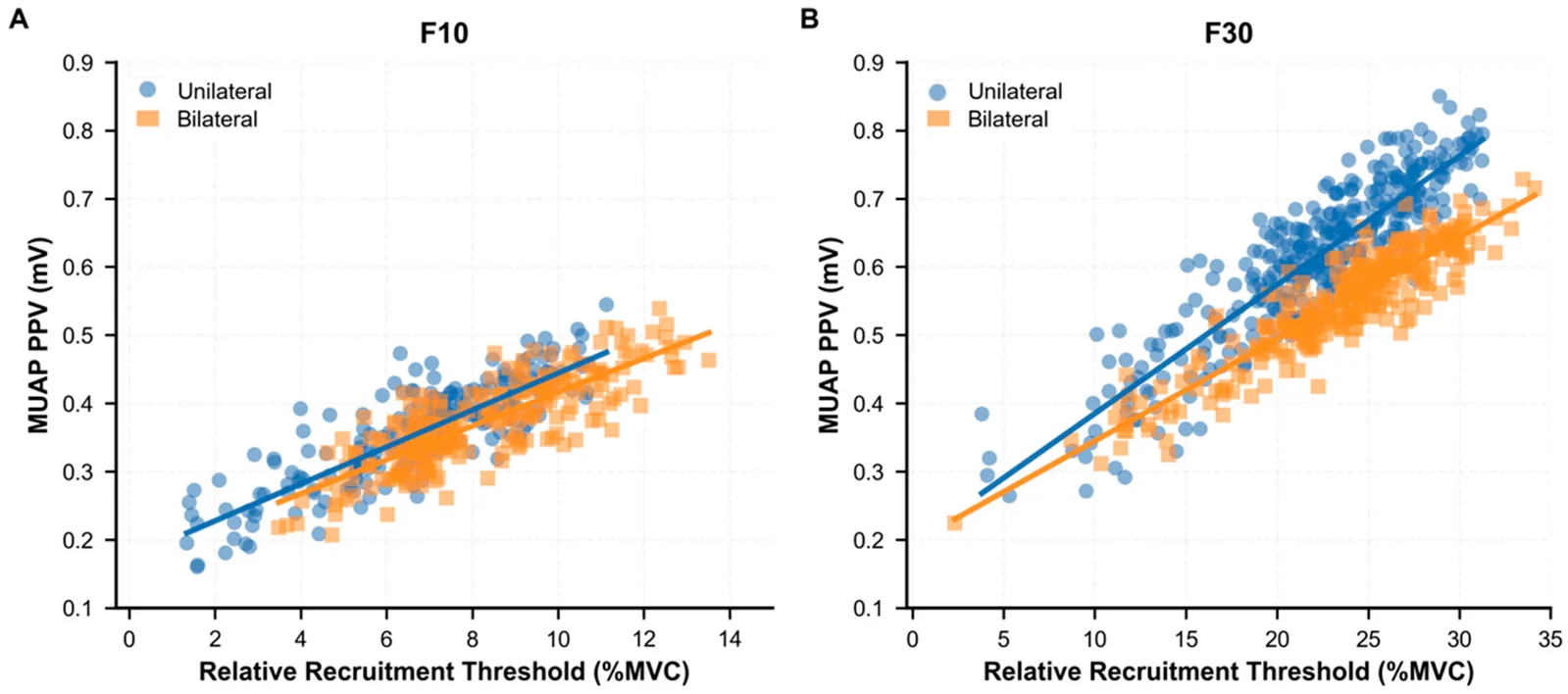
Linear Relationship Between Motor Unit Action Potential Amplitude and Relative Recruitment Threshold. Motor unit action potential peak-to-peak voltage (MUAP_PPV, mV) plotted against relative recruitment threshold (%MVC) across force levels and contraction sides. (**A**) F10 force level contraction condition showing separate linear regressions for bilateral (blue circles and line) and unilateral (orange squares and line) contractions. (**B**) F30 force level contraction condition with the same bilateral and unilateral comparison. Individual motor units are represented by scatter points, with fitted regression lines indicating the linear relationship within each condition. All correlations were statistically significant (*p* < 0.001). R^2^ values ranged from 0.669–0.832 across conditions, indicating that 66.9–83.2% of the variance in MUAP amplitude was explained by relative recruitment threshold.

For motor unit action potential peak-to-peak amplitude (MUAP_PPV), linear mixed-effects modeling revealed a significant main effect of force level (F30 vs. F10: β = +0.168, SE = 0.008, z = 21.98, *p* < 0.001), whereas the main effect of contraction side was not significant (Unilateral vs. Bilateral: β = −0.014, SE = 0.008, z = −1.81, *p* = 0.070). Linear regression examining the relationship between MUAP_PPV and RT across the four force × side conditions demonstrated significant positive correlations in all conditions (Figure 4; R^2^ range = 0.669–0.832).

Participant-level sensitivity analysis. When the data were aggregated to one equally weighted value per participant, the RT laterality effect remained statistically significant at both force levels (10% MVC: n = 12, Unilateral − Bilateral = −1.51% MVC, 95% CI [−2.42, −0.60], t = −3.64, *p* = 0.004; 30% MVC: n = 14, −1.44% MVC, 95% CI [−2.64, −0.24], t = −2.59, *p* = 0.023), closely matching the model-based contrasts (−1.52 and −1.29% MVC). These findings confirm that the laterality effect was robust to the level of analysis and not attributable to disproportionate contributions from participants with larger numbers of decomposed units.

### 3.3. Motor Unit Discharge Rate

**Figure 5:**
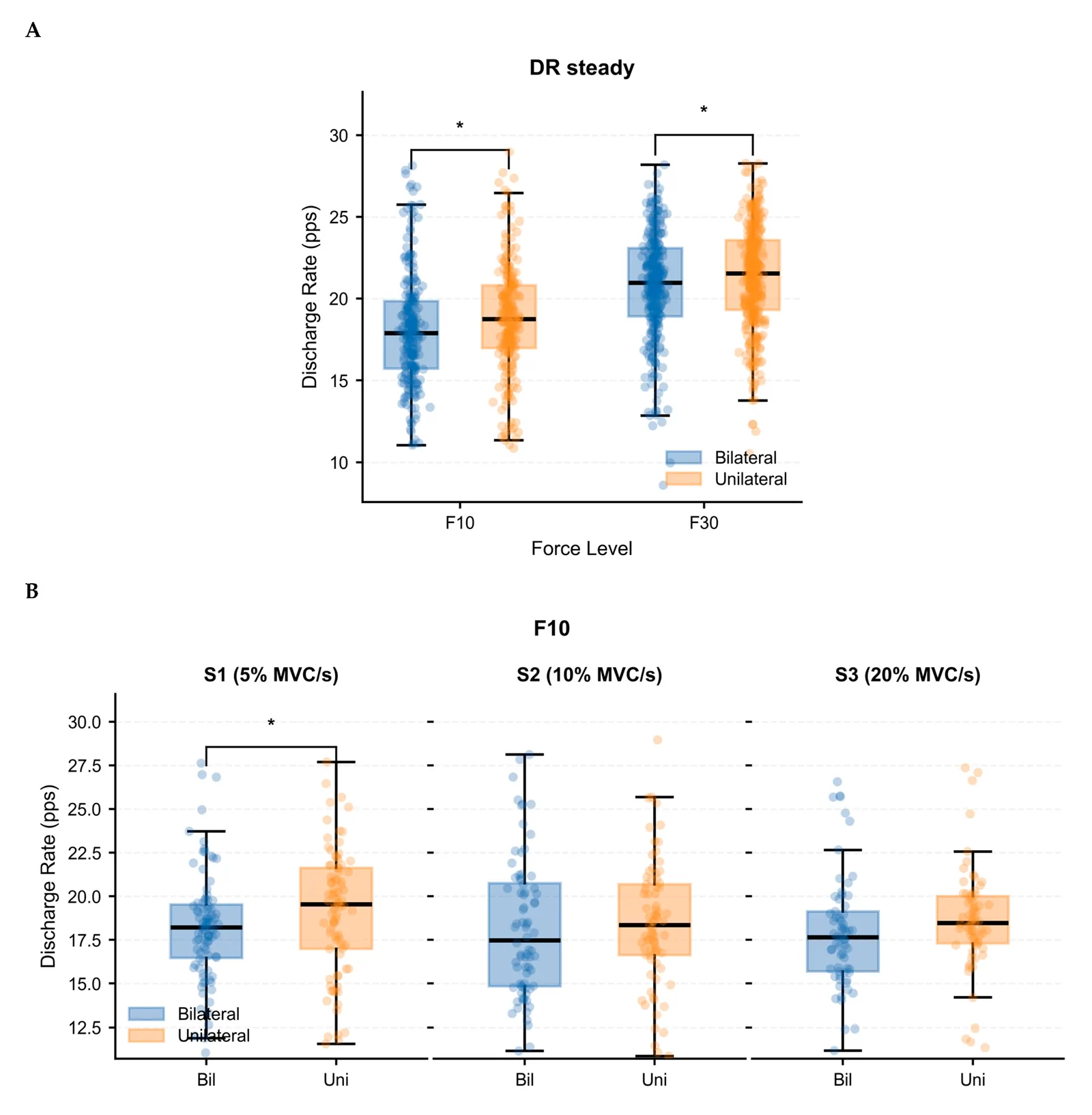

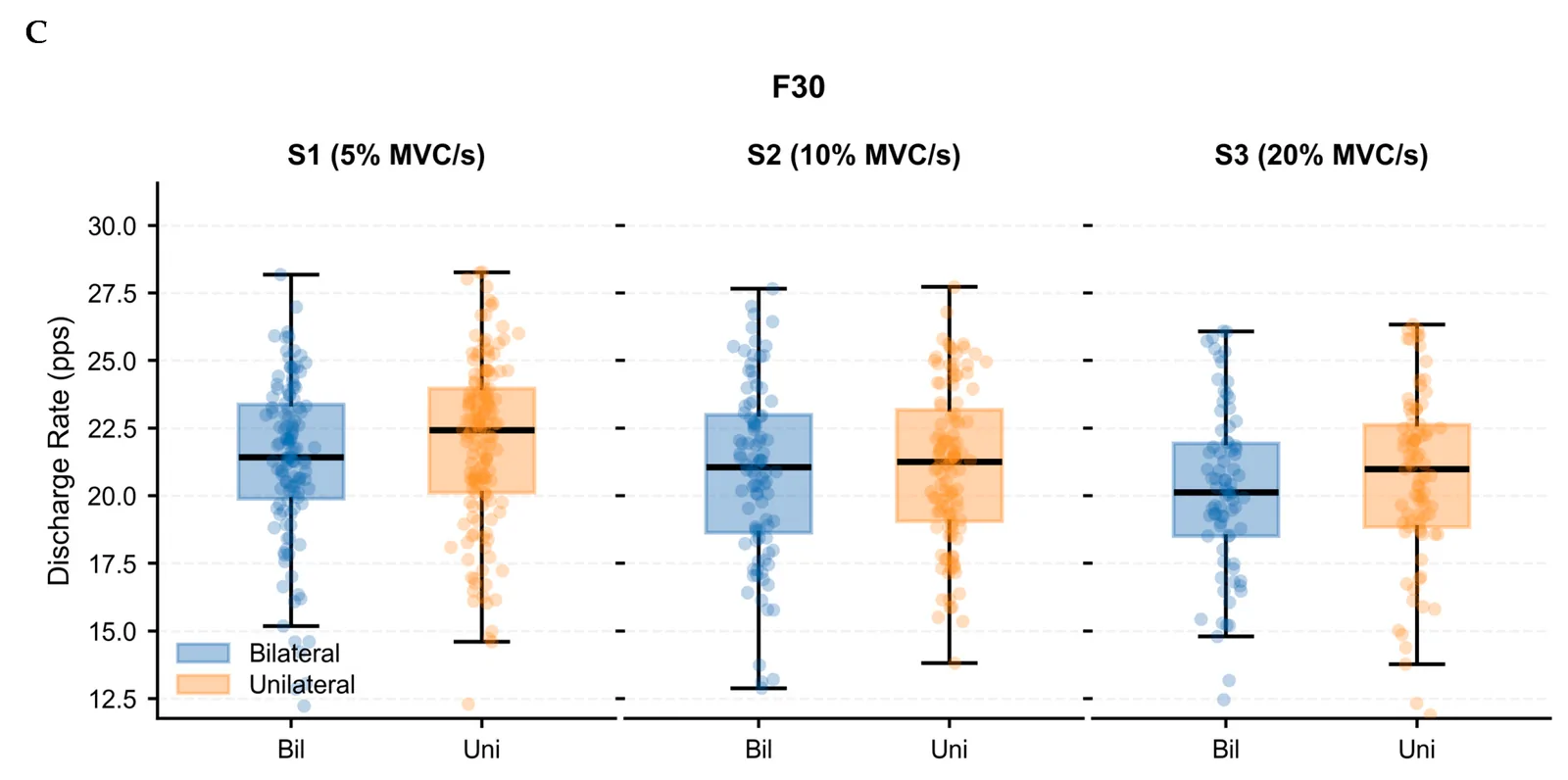
Motor unit discharge rate (DR steady) across force levels and contraction sides. (**A**) Distribution of DR steady at F10 and F30 for bilateral (Bil) and unilateral (Uni) contractions. (**B**) Discharge-rate distributions across the three contraction rates (S1 [5% MVC/s], S2 [10% MVC/s], S3 [20% MVC/s]) during F10 bilateral and unilateral contractions. (**C**) Discharge-rate distributions across the same three contraction rates during F30 bilateral and unilateral contractions. * *p* < 0.05.

Linear mixed-effects modeling revealed a significant main effect of force level on steady-state discharge rate (DR_steady; F30 vs. F10: β = +2.68, SE = 0.206, z = 13.04, *p* < 0.001), with DR_steady significantly higher in the F30 condition (estimated marginal mean = 20.96 pps) than in the F10 condition (18.40 pps; Figure 5A). Contraction side also exerted a significant main effect (Unilateral vs. Bilateral: β = +0.694, SE = 0.205, z = 3.38, *p* < 0.001), with unilateral contractions producing significantly higher DR_steady than bilateral contractions. EMMs for the four force × side conditions were: F10 bilateral 18.06 pps (95% CI [17.41, 18.71]), F10 unilateral 18.74 pps ([18.09, 19.40]), F30 bilateral 20.71 pps ([20.09, 21.32]), and F30 unilateral 21.21 pps ([20.61, 21.81]). Model-based contrasts confirmed higher unilateral discharge at both force levels (Unilateral − Bilateral: F10, +0.68 pps, 95% CI [0.10, 1.27], *p* = 0.022; F30 +0.50 pps, 95% CI [0.004, 1.000], *p* = 0.048).

Regarding interactions, the force × side interaction was not significant (β = −0.18, SE = 0.39, 95% CI [−0.95, 0.59], z = −0.46, *p* = 0.644). The force × force-ramp-rate and side × force-ramp-rate interactions were also not significant, as was the three-way interaction (all *p* > 0.05; Figure 5B,C). These results indicate that the unilateral discharge-rate advantage did not show a significant interaction with force level or force-ramp rate.

Participant-level sensitivity analysis. When the data were aggregated to one equally weighted value per participant, the DR_steady laterality effect remained statistically significant at both force levels (10% MVC: n = 12, Unilateral − Bilateral = +0.74 pps, 95% CI [0.29, 1.19], t = +3.60, *p* = 0.004; 30% MVC: n = 14, +0.47 pps, 95% CI [0.19, 0.75], t = +3.68, *p* = 0.003), closely matching the model-based contrasts (+0.68 and +0.50 pps) and confirming that the effect was robust to the level of analysis.

### 3.4. FCC Signal and Force Variability

**Figure 6:**
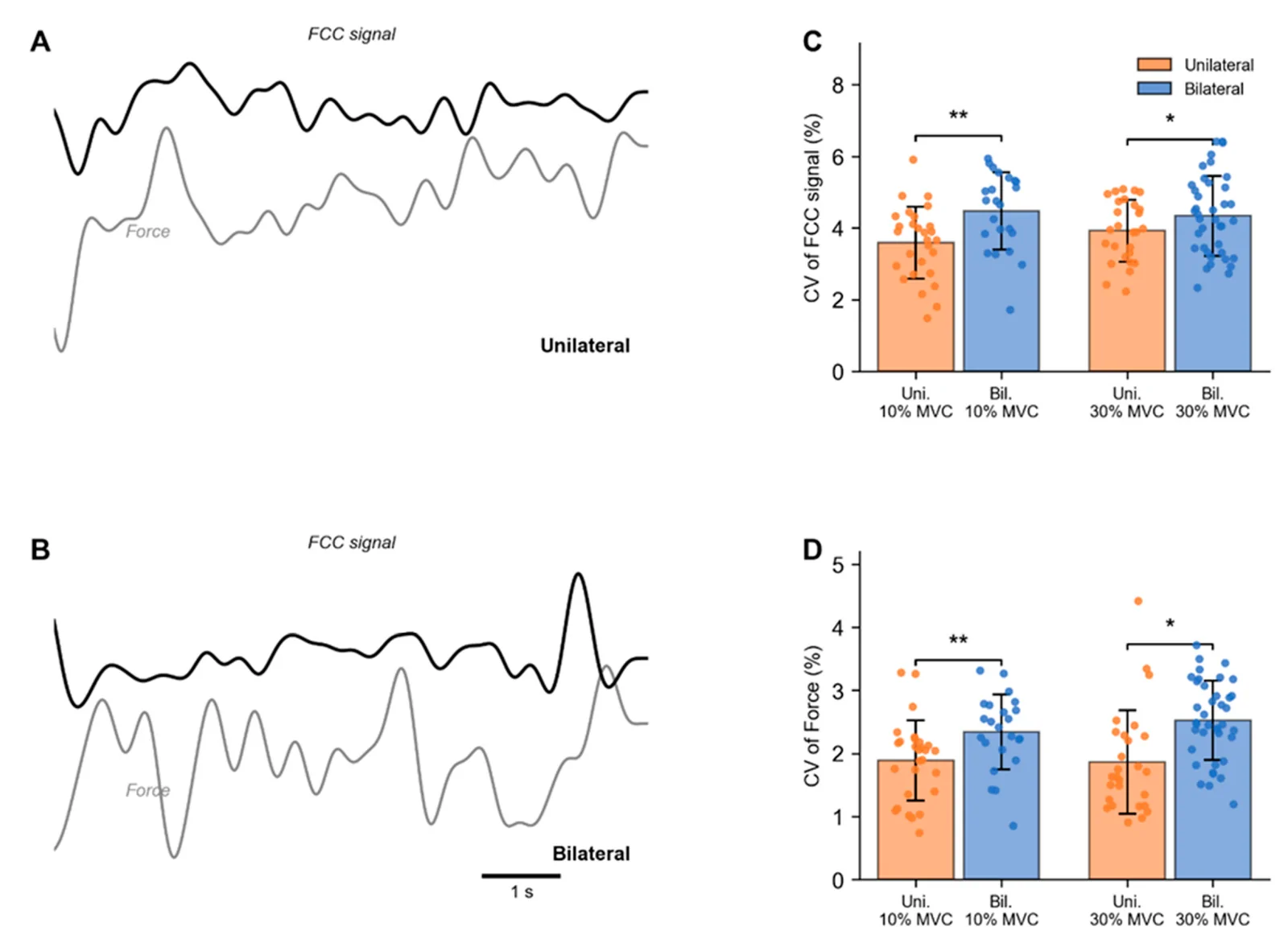
FCC Signal and Force Variability. (**A**,**B**) Representative FCC signal (black) and force output (gray) during unilateral (**A**) and bilateral (**B**) contractions at 30% MVC. Bilateral contractions show greater force fluctuation. (**C**) PC1-CV. Bilateral significantly higher than unilateral (Task effect, *p* < 0.0001). (**D**) Coefficient of variation of force. Bilateral significantly higher than unilateral. * *p* < 0.05; ** *p* < 0.01.

We quantified the relationship between low-frequency neural drive characteristics and force output by computing a proxy for common synaptic input (FCC estimate) from motor unit spike trains. Figure 6A,B illustrate the time-varying FCC signal (black trace) and force signal (gray trace) derived from motor unit discharge rates during representative unilateral and bilateral contractions. Bilateral contractions exhibited greater force fluctuations, and both the FCC and force signals demonstrated similar low-frequency oscillatory characteristics.

The first principal component (PC1) was extracted from motor unit discharge rates via principal component analysis (PCA) and explained 41–45% of the variance in motor unit discharge rates. Specifically, PC1 explained 44.3% ± 6.5% (10% MVC) and 40.4% ± 9.8% (30% MVC) during unilateral contractions, and 44.1% ± 8.6% (10% MVC) and 45.2% ± 7.9% (30% MVC) during bilateral contractions. No significant differences were observed between contraction types (*p* = 0.084 and *p* = 0.131, respectively).

Linear mixed-effects model (LMM) analysis revealed a significant effect of task type on the coefficient of variation of PC1 (PC1-CV; β = −0.86, 95% CI [−1.30, −0.42], *p* < 0.0001). Neither force level nor the interaction term showed significant effects. Bilateral contractions exhibited significantly greater PC1-CV (10% MVC 4.47% ± 1.07%; 30% MVC 4.34% ± 1.11%) compared to unilateral contractions (10% MVC 3.59% ± 1.01%; 30% MVC 3.92± 0.86%; Figure 6C). Similarly, the coefficient of variation of force output (Force CV) showed a significant task type effect (β = −0.41, 95% CI [−0.73, −0.09], *p* = 0.012). Bilateral contractions demonstrated significantly greater Force CV (10% MVC 2.34% ± 0.60%; 30% MVC 2.52% ± 0.63%) compared to unilateral contractions (10% MVC 1.88% ± 0.63%; 30% MVC 1.86% ± 0.82%; Figure 6D).

Both PC1-CV and Force-CV were elevated during bilateral relative to unilateral contractions, indicating greater variability in both the estimated common neural drive (PC1, used here as a proxy for common synaptic input) and force output during bilateral coordination.

**Figure 7:**
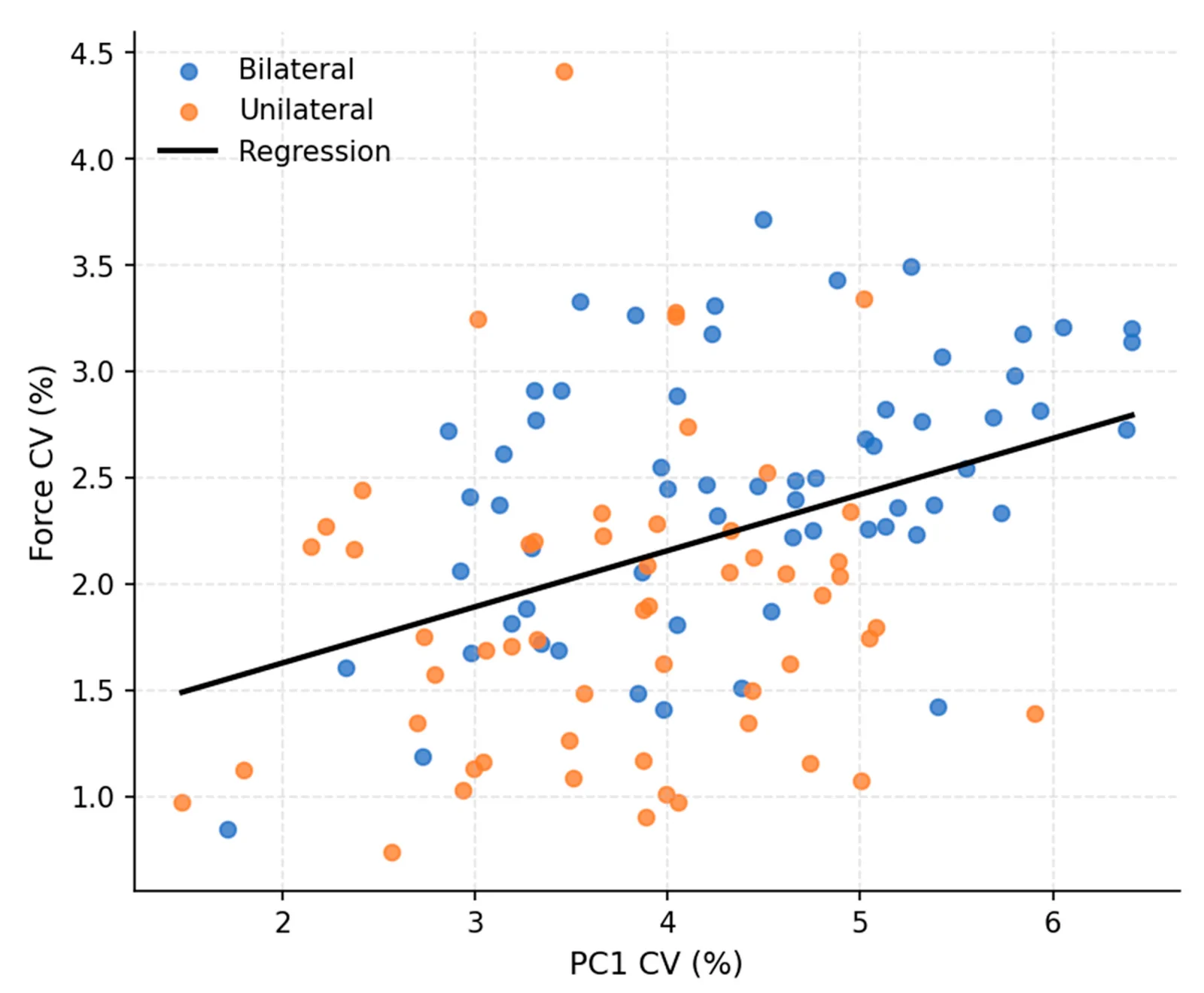
FCC Signal Variability and Force Variability Relationship. Scatter plot of PC1-CV versus force CV (R^2^ = 0.25, *p* < 0.001).

Because trials were nested within participants, we assessed the PC1-CV/Force-CV relationship using analyses that account for within-participant dependence rather than a pooled correlation. To assess this relationship, we applied two complementary approaches: a repeated-measures correlation revealed a significant positive within-participant association (r_rm = 0.53, 95% CI [0.37, 0.65], *p* < 0.001, df = 100), corroborated by a linear mixed-effects model with a by-participant random intercept (β = 0.37, 95% CI [0.25, 0.49], *p* < 0.001; ICC = 0.35). The marginal variance explained by PC1-CV was R^2^ ≈ 0.25, indicating that fluctuations in the common neural drive proxy are one important contributing factor to force steadiness rather than its sole or principal determinant. The majority of force variability remained unexplained and is likely attributable to motor-unit–independent discharge noise, intrinsic muscle mechanical properties, and antagonist co-activation, which were not assessed in the present study, Figure 7.

## 4. Discussion

### 4.1. The Interlimb Effect Shows No Significant Modulation by Force Level or Rate of Force Development

Using an asymmetric bilateral force-tracking paradigm, the present study systematically examined the influence of the interlimb effects on motor unit (MU) properties of the biceps brachii across two force levels (10% and 30% MVC) and three rates of force development (5%, 10%, and 20% MVC/s). The principal findings were as follows: under bilateral conditions, recruitment thresholds (RT) were elevated, steady-state discharge rates (DR) were reduced, PC1-CV was increased, and Force-CV was increased, whereas the proportion of total variance explained by PC1 remained unaffected. In the linear mixed-effects models, all of these changes manifested as robust main effects of condition (bilateral vs. unilateral), while main effects of force level, force × condition interactions, and rate-related interactions were uniformly non-significant.

These findings diverge in important ways from those of prior studies. Bao et al. [21] reported reduced discharge rates and elevated low-frequency oscillations during symmetric constant-force bilateral isometric contractions; however, since rate of force development was not manipulated in that design, the question of whether the interlimb effects varies with rate could not be addressed. Oda and Moritani [37] documented reduced bilateral neural drive at maximal voluntary contraction, but the neural control strategies underlying maximal contractions are fundamentally distinct from those governing submaximal dynamic tracking, and the extent to which their conclusions generalise to the latter context remains uncertain. The fully crossed design of the present study, two force levels combined with three rate levels, afforded a systematic test of the task-dependence of the interlimb effect. The absence of any interaction (bilateral–unilateral RT difference stable across force levels; the force × side interaction was non-significant, β = +0.23, *p* = 0.623) indicates that, within the force–rate space examined here, the interlimb effect appeared decoupled from these task parameters and may represent an intrinsic property of the asymmetric bilateral coordination constraint. Furthermore, whereas the majority of prior research has employed symmetric isometric paradigms, the asymmetric dynamic tracking design adopted in the present study more closely approximates the demands of real-world bilateral movement, in which each limb executes a distinct force task. This provides new experimental evidence for understanding the neural constraint mechanisms operative in ecologically valid bilateral motor tasks.

### 4.2. Cortical Versus Spinal Contributions to the Interlimb Effect

Bilateral and unilateral movements are encoded differently at the cortical level. Bilateral tasks elicit stronger and more widespread activation in the primary motor cortex (M1) and supplementary motor area [38,39], and individual M1 neurons exhibit selective discharge patterns for each task type [3,40]. One might therefore hypothesise that the interlimb effects observed in the present study reflects differential cortical command signals. However, the interaction analyses impose critical constraints on this interpretation.

If the magnitude of the interlimb effects were governed by dynamic cortical adjustments to task difficulty, one would expect that the increased cortical engagement from F10 to F30 would correspondingly alter the processing of bilateral coordination constraints, yielding a significant force × condition interaction. The data do not support this prediction. Although absolute RT increased substantially from F10 to F30 (approximately 7.5% to 22.8% MVC), the bilateral–unilateral RT difference remained virtually unchanged; similarly, the bilateral–unilateral differences in DR, PC1-CV, and Force-CV were highly consistent across force levels. The cortical encoding of force level is precise and continuously scaled with output force [41], yet the interlimb effects was entirely unresponsive to this variation. This dissociation is consistent with the possibility that the constraint resides downstream of the cortical command, at the level of synaptic integration within the motoneuron pool, although a cortical or mixed contribution cannot be excluded from the present non-invasive data.

Enhanced cortical activation does not necessarily translate into increased net excitatory output to the target muscle. The broader cortical activity observed during bilateral tasks partly reflect additional processing demands, such as interhemispheric coordination and bilateral sensory integration, rather than augmented descending drive per se. Descending signals undergo a further stage of integration at the spinal level before ultimately acting on motor units [41]. We hypothesise that the stable proprioceptive afference generated by the contralateral limb maintaining a sustained 20% MVC contraction may impose a relatively stable net inhibitory bias on the ipsilateral motoneuron pool via spinal inhibitory interneuron circuits [42]. This bias acts at the level of synaptic integration and its magnitude does not vary with changes in the force command to the ipsilateral limb, would naturally producing a bilateral–unilateral difference of consistent magnitude across force levels. This interpretation is consistent with the reduced bilateral neural drive reported by Oda and Moritani [37] at maximal contraction, and extends that observation to the submaximal dynamic tracking domain. We emphasise, however, that the present study did not directly measure spinal inhibitory circuitry, corticospinal excitability, or afferent input; the proposed spinal locus therefore represents one possible explanation inferred from behavioural data rather than a demonstrated mechanism.

### 4.3. Preservation of the Size Principle and Reorganisation of Motor Unit Recruitment Properties

Henneman’s size principle predicts that motor units are recruited in an orderly sequence according to their size [43]. The ordinal recruitment sequence remained consistent with the size principle across all conditions in the present study. The correlation between MU action potential peak-to-peak voltage (MUAP_PPV) and relative RT was strong in all four force × condition combinations (R^2^ = 0.669–0.832). This indicates that the sequential organisation was consistently preserved under both unilateral and bilateral conditions.

In this organisation, smaller motor units are activated at low thresholds. Larger units are activated at higher thresholds. These findings are in agreement with recent reports from high-density surface EMG studies of large MU samples in lower-limb muscles [44] and upper-limb dynamic tracking tasks [26]. It should be noted, however, that MUAP_PPV is influenced by electrode position, subcutaneous tissue conductivity, muscle-fibre geometry, and detection bias, and therefore cannot be treated as a direct measure of the anatomical size of a motor unit; we accordingly interpret the MUAP_PPV–RT relationship as evidence that the ordinal recruitment sequence was preserved, rather than as direct confirmation of anatomical recruitment order. MUAP_PPV did not differ significantly between bilateral and unilateral conditions (*p* = 0.070), whereas RT was significantly elevated and DR was significantly reduced. Two mechanistic pathways could, in principle, account for this pattern. First, the bilateral condition may activate a subpopulation of motor units whose physical size is similar to those recruited unilaterally but whose excitability state differs. MUAP_PPV is thought to reflect chiefly the anatomical size of the motor unit (muscle fibre cross-sectional area) and its geometric relationship to the recording electrode; it would be insensitive to the neural excitability state of the unit. Consequently, even if bilateral constraints alter the specific subpopulation of units preferentially recruited, MUAP_PPV may remain unchanged provided the size distribution of the activated pool is similar to that observed during unilateral contraction [41]. This is consistent with task-dependent motor unit subpopulation selection described in animal studies [45] and human fine motor control research [46]. Second, the same complement of motor units may undergo a global downward shift in excitability under the net inhibitory afference associated with bilateral coordination, resulting in elevated RT and reduced DR in response to an equivalent net descending drive. Under this scenario, the pool composition need not change; rather, the response gain of individual units to synaptic input is altered. Hug et al. [47] in examining the non-uniform distribution of inhibitory inputs to motor units, demonstrated that presynaptic inhibition and Ib inhibitory pathways can selectively modulate the excitability of different units within the motoneuron pool without altering their morphological characteristics, providing a candidate mechanistic reference for the dissociation between MUAP_PPV and recruitment properties observed here.

Under either account, the elevation in RT need not reflect a change in the physical size of recruited motor units, but rather a change in their excitability state or in the composition of the activated subpopulation. The coexistence of unchanged MUAP_PPV and elevated RT reflects a state in which the ordinal sequence prescribed by the size principle is preserved, but the excitability calibration of the recruitment process is reset. Crucially, this reorganisation of recruitment properties was equally consistent across force levels, in accordance with the overall force-invariance of the interlimb effects, and provides motor-unit-level evidence compatible with, though not proof of, the spinal-constraint interpretation. To maintain the target force output despite reduced discharge rates, the system must compensate through recruitment adjustments in MU pool composition can offset reductions in discharge rate to sustain force output [48].

### 4.4. Coupling Between Low-Frequency Common Drive Variability and Force Stability

The motoneuron pool receives both common and independent synaptic inputs from supraspinal and spinal sources. Low-frequency common input (<5 Hz; the δ band) is the principal neural determinant of force output stability during isometric tasks [29]. In the present study, the PC1 signal extracted from MU discharge trains via principal component analysis serves as a proxy for low-frequency common drive to the motoneuron pool. PC1-CV was significantly elevated under bilateral conditions (*p* < 0.0001), whereas the proportion of total variance explained by PC1 remained stable across conditions (41–45%), indicating that the overall synchronisation structure of the low-frequency drive was not reorganised, but that its temporal fluctuations increased. Because trials were nested within participants, we assessed the PC1-CV / Force-CV relationship using analyses that account for within-participant dependence rather than a pooled correlation.

A repeated-measures correlation revealed a significant positive within-participant association (r_rm = 0.53, 95% CI [0.37, 0.65], *p* < 0.001), corroborated by a linear mixed-effects model with a by-participant random intercept (β = 0.37, 95% CI [0.25, 0.49], *p* < 0.001; ICC = 0.35). The marginal variance explained by PC1-CV was R^2^ ≈ 0.25. This indicates that temporal variability in the common-drive proxy is one important contributing factor to force fluctuation rather than its sole or principal determinant; the majority of force variability remained unexplained and is likely attributable to recruitment–derecruitment dynamics, MU-independent discharge noise, and downstream factors including intrinsic muscle mechanical properties and musculotendinous transmission, which were not assessed here. This magnitude is broadly comparable to the pCSI–Force association reported by Borzuola et al. [31] in the tibialis anterior. Cabral et al. [49] further showed that muscle contractile properties can modulate the transmission efficiency of common spinal input, implying that the influence of PC1-CV on Force-CV may vary across muscles. Because bilateral and unilateral conditions tracked identical force trajectories, the elevation in Force-CV under bilateral conditions cannot be attributed to differences in task demand and is more plausibly related to the neural variability observed under the bilateral constraint.

The critical finding in this context is that the elevations in both PC1-CV and Force-CV manifested exclusively as significant main effects of condition, with no main effect of force level and no interaction effects (F30 data: PC1-CV bilateral 4.34% ± 1.11% vs. unilateral 3.92% ± 0.86%; Force-CV bilateral 2.52% ± 0.63% vs. unilateral 1.86% ± 0.82%, a pattern consistent with that observed at F10). This consistency across force levels carries important implications: if the decline in force stability were merely a secondary consequence of elevated cognitive–motor load at higher force levels, one would expect an incremental worsening with increasing force. In contrast, the bilateral constraint-induced increase in variability was of comparable magnitude at both low and high force levels. This is consistent with the interpretation that elevated low-frequency drive variability constitutes a force-level-independent manifestation of the interlimb effects at the spinal level. This finding aligns with the conclusions of Feeney et al. [50], who reported that common synaptic input variability independently modulates force stability. Furthermore, our results extend their findings to the context of bilateral coordination constraints. The foregoing observations can be integrated within a corticospinal hierarchical framework. At the cortical level, bilateral tasks engage a distinct pattern of descending drive relative to unilateral tasks [39]. At the spinal level, these descending signals are integrated with proprioceptive afference generated by sustained contraction of the contralateral limb [42], potentially yielding a net inhibitory bias that is insensitive to force level—manifesting as upward shifts in RT and reduced effective drive. At the motor unit level, the ordinal sequence prescribed by the size principle is preserved, but the excitability calibration of the activated pool or its subpopulation composition is altered [47]. At the force output level, greater variability in the common-drive proxy was associated with poorer force stability [31,49]. We stress that the central support for this framework, the absence of significant force- or rate-dependent modulation of the interlimb effect, provides indirect evidence only; localisation of the constraint to the spinal-integration level remains one interpretation consistent with the data and awaits confirmation with direct neurophysiological measurement.

### 4.5. Limitations

First, the sample was small (n = 14) and demographically narrow, comprising young adults (18–22 years) who were predominantly men. No a priori statistical power (sample-size) calculation was performed; the study was designed as a preliminary, exploratory investigation, and the sample size was determined pragmatically on the basis of feasibility and the intensive within-participant, multi-condition high-density EMG protocol. The characteristics of the interlimb effect may well differ across these populations, and confirmation in larger, sex-balanced, and demographically broader samples is required. In particular, the two women in our sample were too few to permit any sex-disaggregated analysis. Male and female motor-unit behaviour cannot be assumed to be identical under bilateral control or fatigue, given documented sex differences in fibre-type composition, discharge behaviour, and fatigue resistance. For instance, contralateral effects of fatiguing contractions are largely mediated by central (supraspinal) mechanisms, and sustained maximal contractions produce greater central fatigue and a more pronounced ‘cross-over’ of force and voluntary-activation deficits to the contralateral limb in men than in women. In addition, women generally exhibit greater fatigue resistance during sustained isometric contractions at comparable relative intensities—attributable in part to sex differences in muscle mass, fibre-type composition, and neuromuscular activation—and typically show higher motor-unit discharge rates at submaximal force outputs. Therefore, sex should be treated as a potential moderator of the interlimb effect, but the present design was not powered to resolve this question. The large number of motor units analysed (*N* ≈ 1111) reflects repeated within-participant sampling and does not increase participant-level statistical power. Accordingly, the present findings are preliminary, and the generalisability of the present conclusions to these populations awaits investigation in adequately powered, sex-balanced samples.

Second, we did not detect significant force- or rate-dependent modulation of the interlimb effect was found only within the range of 10–30% MVC and rates of force development of 5–20% MVC/s. Whether these conclusions generalise to higher force levels, such as those approaching maximal voluntary contraction, or to faster dynamic tracking conditions remains unclear. From a physiological standpoint, cortical drive saturation and full recruitment of the spinal motoneuron pool may occur at high force levels. These conditions may alter the expression of bilateral constraints. However, whether the invariance identified here persists under more extreme task parameters requires dedicated experimental investigation.

Third, the contralateral constraint limb (left arm) maintained a fixed 20% MVC isometric contraction throughout, representing a standardised experimental control. However, bilateral motor coordination in naturalistic settings is considerably more complex, with the contralateral limb typically also in a dynamic state. Whether the intensity and dynamic characteristics of contralateral-limb contraction modulate the magnitude of the interlimb effect cannot be determined from the present data. In addition, we did not measure the thickness of the biceps subcutaneous adipose tissue (e.g., by ultrasonography or skinfold), which acts as a volume conductor that attenuates and spatially low-pass–filters the surface signal; inter-individual variation in adipose thickness may therefore have influenced which motor units were detectable and could bias the retained sample toward more superficial units. Accordingly, our sample should be regarded as representative of the detectable superficial motor-unit population rather than of the entire motoneuron pool.

Fourth, the MU sample captured by high-density surface EMG is concentrated in the superficial muscle belly, and the detection rate of low-discharge-rate or deeply situated motor units is limited by signal-to-noise constraints. Whether the changes in recruitment properties observed here are uniformly present across the full motoneuron pool, including deep and high-threshold units, awaits verification using techniques with greater spatial resolution, such as intramuscular EMG or ultrasound-guided fine-wire recording.

Finally, the mechanistic interpretation advanced here, that spinal inhibitory interneuron circuits impose a stable inhibitory bias on the ipsilateral motoneuron pool, rests on indirect inference from behavioural (EMG and force) data. The present study did not measure H-reflexes, motor-evoked potentials, corticospinal excitability, or spinal interneuronal activity. Consequently, the specific contribution of spinal inhibition cannot be isolated. The proposed spinal localisation should be regarded as one plausible interpretation. This interpretation should be tested directly in future work, for example, using H-reflex conditioning, transcranial magnetic stimulation, or intramuscular recordings. Likewise, the PC1-CV / Force-CV association constitutes observational evidence and is insufficient in itself to establish causality.

### 4.6. Tentative Implications for Rehabilitation

The following implications are offered as hypotheses rather than clinical recommendations, because the present participants were healthy young adults performing an artificial isometric force-tracking task rather than a clinical rehabilitation intervention. Bilateral symmetric training is commonly employed in rehabilitation to harness the “facilitory” influence of the intact limb on the recovery of the affected limb, with theoretical support from the stronger cortical activation elicited by bilateral tasks. Our data raise the following hypothesis. Under bilateral coordination constraints, greater cortical activation may not translate into greater effective spinal drive to the target muscle, as suggested by the elevated recruitment thresholds and reduced discharge rates observed here. Additionally, the bilateral constraint introduces extra force variability that is independent of force level, which could be relevant when precise force control is the training goal. These possibilities suggest that unilateral task-specific training might afford more favourable neural-drive conditions during early stages aimed at establishing stable force control, and that the optimal timing and loading parameters for introducing bilateral training warrant dedicated study. Because the spinal circuitry of patients with neuromuscular disease or central-nervous-system injury differs fundamentally from that of healthy individuals, none of these conjectures should be extrapolated to clinical populations without direct investigation in the relevant patient groups.

## 5. Conclusions

The present study used an asymmetric bilateral dynamic force-tracking paradigm. We provide motor-unit-level evidence for the systematic influence of bilateral coordination constraints on the neural drive to the biceps brachii. Specifically, recruitment thresholds were elevated, steady-state discharge rates were reduced, low-frequency common drive variability was increased, and force output stability was degraded. These effects were consistent across two force levels (10% and 30% MVC) and three rates of force development (5%, 10%, and 20% MVC/s), with force-related and rate-related interaction effects uniformly non-significant. Within the force and rate range examined, this invariance indicates that the interlimb effect was not dynamically rescaled with task demand. We emphasise, however, that this behavioural observation is compatible with, but does not by itself distinguish between, a stable constraint acting at the level of synaptic integration within the spinal motoneuron pool and a corticospinal or brainstem bias that is simply not rescaled with task parameters. One candidate account is that sustained contraction of the contralateral limb generates afferent activity. This activity might impose a relatively stable inhibitory bias on the ipsilateral motoneuron pool. We advance this account as a hypothesis rather than as a finding, as the involvement of spinal interneuron circuits is inferred from the behavioural pattern and was not measured here.

The orderly, size-related recruitment sequence was preserved under all conditions; the strong correlation between MUAP_PPV and RT is consistent with a preserved recruitment ordering under bilateral constraints, while suggesting that the excitability calibration of the recruited pool, or the composition of the activated subpopulation, is altered. This inference should be read with the caveat that MUAP_PPV is influenced by electrode position, tissue conductivity, muscle-fibre geometry and detection bias, and therefore does not constitute a direct measure of motor unit anatomical size.

After accounting for the repeated observations within participants, the association between PC1-CV and Force-CV remained significant. This indicates that variability in the common neural drive is one important contributing factor to force instability. However, approximately three-quarters of the variance in force stability remained unexplained. This unexplained variance is likely attributable to uncorrelated motor unit discharge noise, muscle mechanical properties, and antagonist co-activation, none of which were measured in the present study.

This association likewise exhibited no dependence on force level. Collectively, these findings indicate that asymmetric bilateral coordination constraints impose a task-parameter-insensitive neural bias whose neural locus cannot be resolved from the present behavioural data. Because the study did not measure cortical, corticospinal, or spinal neurophysiological variables (H-reflexes, presynaptic inhibition, or TMS-evoked potentials), any hierarchical localisation of this bias remains an interpretation to be tested with targeted neurophysiological methods rather than a result demonstrated by the present experiment. These conclusions are further restricted to the population studied. This population consisted of healthy young adults aged 18–22 years, who were predominantly men and performed an artificial isometric force-tracking task. Therefore, our findings should not be extrapolated to women, older adults, trained athletes, or individuals with neuromuscular disorders without dedicated investigation. Within these boundaries, the study provides new experimental evidence relevant to understanding the neural constraints operating in bilateral motor tasks. It also raises mechanistically important questions regarding the design of neuromuscular rehabilitation strategies centred on bilateral coordination training.

## Data Availability

The datasets used and/or analyzed during the current study are available from the first and corresponding authors upon reasonable request.

## Supplementary Materials

The following supporting information can be downloaded at: https://www.mdpi.com/article/10.3390/life16091404/s1, Table S1:Motor Unit Count by Contraction Side; Table S2:Motor Unit Count by Force Level; Table S3:Motor Unit Count by Contraction Velocity; Table S4:Summary of Statistical Tests on Motor Unit Count; Table S5:ACCURACY & COVisi_steady Statistics by Side.
