# Supplementary material for: Motor Unit Behaviour in the Biceps Brachii During Asymmetric Bilateral Versus Unilateral Dynamic Force Tracking Across Force Levels and Rates of Force Development: A Preliminary Study
Source: Life (Basel). 2026 Aug 25;16(9):1404. doi: 10.3390/life16091404 (PMC13608433; doi:10.3390/life16091404)
Supplement: Supplementary file 1 [file life-16-01404-s001.zip › life-4487459-supplementary.pdf]

Supplementary Material

Motor Unit Decomposition Statistics

Table S1. Motor Unit Count by Contraction Side

Side	Total MU	Mean ± SD	n
Bil	533	38.07 ± 14.24	14
uni	578	41.29 ± 14.75	14
Total	1111	39.68 ± 14.32	14

Table S2. Motor Unit Count by Force Level

Force	Total MU	Mean ± SD	n
F10	461	38.42 ± 20.70	12
F30	650	46.43 ± 12.62	14
Total	1111	42.73 ± 16.97	14

Table S3. Motor Unit Count by Contraction Velocity

Velocity	Total MU	Mean ± SD	n
V5	447	31.93 ± 12.46	14
V10	364	26.00 ± 10.13	14
V20	300	23.08 ± 9.25	13
Total	1111	27.10 ± 11.11	14

Table S4. Summary of Statistical Tests on Motor Unit Count

Factor/Effect	Condition 1	Condition 2	Test Statistic	p-value
Side (main)	Bilateral (38.07±13.72)	Unilateral (41.29±14.21)	t(26) = -0.587	0.563
Force (main)	F10 (38.42±19.82)	F30 (46.43±12.16)	t(24) = -1.211	0.238
Velocity (main)	V5 (31.93±12.01)	V10 (26.00±9.76) / V20 (23.08±8.89)	F(2,38) = 2.402	0.104
Force within Bilateral	F10 (19.33±9.94)	F30 (21.50±6.11)	t(24) = -0.653	0.520
Force within Unilateral	F10 (19.08±10.10)	F30 (24.93±6.39)	t(24) = -1.719	0.099
Side within F10	Bilateral (19.33±9.94)	Unilateral (19.08±10.10)	t(22) = 0.059	0.954

Table S5. ACCURACY & COVisi_steady Statistics by Side

Side	Accuracy SIL (mean±std)	COVisi_steady (mean±std)	n
Bil	0.9277±0.0195	24.4771±3.3937	533
uni	0.9294±0.0200	24.4578±3.5987	578
